# The Integrated Resource for Reproducibility in Macromolecular Crystallography: Experiences of the first four years

**DOI:** 10.1063/1.5128672

**Published:** 2019-11-22

**Authors:** Marek Grabowski, Marcin Cymborowski, Przemyslaw J. Porebski, Tomasz Osinski, Ivan G. Shabalin, David R. Cooper, Wladek Minor

**Affiliations:** 1 Department of Molecular Physiology and Biological Physics, University of Virginia, Charottesville, Virginia 22908, USA; 2 Center for Structural Genomics of Infectious Diseases (CSGID), University of Virginia, Charottesville, Virginia 22908, USA

## Abstract

It has been increasingly recognized that preservation and public accessibility of primary experimental data are cornerstones necessary for the reproducibility of empirical sciences. In the field of molecular crystallography, many journals now recommend that authors of manuscripts presenting a new crystal structure should deposit their primary experimental data (X-ray diffraction images) to one of the dedicated resources created in recent years. Here, we describe our experiences developing the Integrated Resource for Reproducibility in Molecular Crystallography (IRRMC) and describe several examples of a crucial role that diffraction data can play in improving previously determined protein structures. In its first four years, several hundred crystallographers have deposited data from over 5200 diffraction experiments performed at over 60 different synchrotron beamlines or home sources all over the world. In addition to improving the resource and curating submitted data, we have been building a pipeline for extraction or, in some cases, reconstruction of the metadata necessary for seamless automated processing. Preliminary analysis indicates that about 95% of the archived data can be automatically reprocessed. A high rate of reprocessing success shows the feasibility of using the automated metadata extraction and automated processing as a validation step for the deposition of raw diffraction images. The IRRMC is guided by the Findable, Accessible, Interoperable, and Reusable data management principles.

## I. INTRODUCTION

In recent years, serious concerns have emerged over the reproducibility of biomedical research, triggered by reports pointing out that a significant fraction of findings published in reputable scientific journals could not be replicated by other researchers.^1,2^ There may be multiple reasons why some biomedical experiments cannot be reproduced.^3–5^ However, if the primary data (raw data and metadata) produced by the original experiment have not been preserved, investigation of the source of irreproducibility may be impossible. Similarly, if the primary data are available but not public, the ability to evaluate the reproducibility of an experiment will be limited. The increased awareness of the importance of the public accessibility of primary experimental data was a major factor that led science funding bodies and some journals to shift to open science policies encompassing open access not only to results but also to data.^3^ Currently, most scientists and science-funding agencies consider the preservation of primary data and opening them to public scrutiny as necessary conditions for enabling the reproducibility of experimental sciences. A consensus has emerged that access to scientific data should be governed by the Findable, Accessible, Interoperable, and Reusable (FAIR) principles.^4^

The process of macromolecular structure determination via X-ray crystallography generates a diverse set of experimental and computational data—in short, “the science is in the data.”^5^ Usually, the experimental steps start with cloning, expressing, and purifying the protein, proceed through crystallization, and culminate in an X-ray diffraction experiment. Subsequent steps of structure determination are essentially a computational analysis of the collected data. While good records should be gathered at each step of the structure determination process, data collected in the X-ray diffraction experiment are of essential importance because they are the starting point of the computational process leading to the structural model. These models are often used as the starting point for structural dynamics studies, although time-resolved protein crystallography is its own source of multiple related structures.

Ideally, the diffraction data should have the best quality achievable under the circumstances, providing the highest resolution and completeness while minimizing radiation damage. In practice, these goals can sometimes be at odds with each other. Even at the most advanced beamlines, data collection should be approached thoughtfully and not regarded as a routine technical procedure.^6^ Typically, determining the structure for a single protein requires testing many crystals and results in multiple sets of diffraction images (ranging anywhere from one to thousands of datasets, corresponding to hundreds to millions of individual images). However, usually only a subset of these datasets will be used for determination of the final structural model. It is the preservation of these final subsets of primary data that allows for an independent reprocessing and independent validation and, in some cases, improvement of the resulting model. Our experience shows that asking the deposit (paper) authors or synchrotron facilities for diffraction data that led to the structure determination frequently failed, not because of their ill will but rather for the same reasons why looking for a bag full of jewelry lost in a large landfill is very rarely successful.

Crystallographers have long supported the preservation and sharing of primary diffraction data used in the process of crystallographic structure determination, but technical and financial considerations necessitated the compromise of limiting the data being archived to the highly processed and greatly reduced in size structure factor files. Although these files allow comparison of the model with an electron density map and will remain a vital part of structure deposition, it is important to remember that the data reduction process discards some information and is sometimes performed in a suboptimal way. To address the growing realization that primary crystallographic data should be preserved in their entirety, in 2011 the International Union of Crystallography (IUCr) set up the Diffraction Data Deposition Working Group,^7^ which summarized the reasons for archiving diffraction data and metadata and set forth recommendations for desirable practices.

In addition to enhancing the reproducibility of structural studies, the availability of diverse diffraction data offers several other benefits:^8–10^ it helps to develop and test new crystallographic software by providing training sets for new programs, it helps safeguard data in cases of closures of individual labs and large-scale programs, and it allows for the continuous improvement of the quality of the structural models already deposited in the Protein Data Bank (PDB).^11–16^ In 2019, leading crystallographic journals recommended in a joint editorial^17–20^ that authors submitting manuscripts that report a new structural model or a new method should also provide a Digital Object Identifier (DOI) to the primary diffraction data used to obtain the model or to test the method. For papers describing new crystallographic methods, the editorial recommends making the data available to referees. Furthermore, the editorial postulates that deposition of diffraction data should become mandatory in the future. Implementation of these steps will provide an impulse to “maintain crystallography at the forefront of the effort for enhancing transparency and reproducibility of scientific results.”^17–20^

Until recently, researchers who wanted to share their diffraction data had very limited possibilities—there were no public resources that could accommodate and share large amounts of diffraction data. Following in the footsteps of pioneering initiatives such as MyTARDIS at the Australian Synchrotron^21^ and the raw data collection at the University of Utrecht,^22^ two large-scale resources dedicated to archiving and sharing primary crystallography data came on-line in 2015: SBGRID^20^ and Integrated Resource for Reproducibility in Molecular Crystallography (IRRMC).^23^ In addition, several general-purpose repositories for scientific data, such as Zenodo^24^ and Figshare,^25^ have also gathered some diffraction data. As of August 2019, the IRRMC contained over 8500 datasets from 5200+ diffraction experiments associated with 3800+ individual structures, while SBGRID gathered 525 datasets from 460 structures. This paper describes our experiences in developing and operating the IRRMC and our preliminary analysis of image reprocessing and structure redetermination.

## II. DATA ACQUISITION AND ORGANIZATION

While the IRRMC officially started in 2015, its origins reach to the archive of diffraction images gathered throughout the last 20+ years by crystallographers in Minor Lab at the University of Virginia. The archive contained all the diffraction datasets collected by the lab and was organized in a system of computer directories according to projects, collection dates, and beamlines where data were collected. It was stored on a shared network drive, accessible to all researchers in the lab and to some collaborators. The archive was subsequently extended by diffraction images gathered in the course of the activities of several structural genomics (SG) programs, in which Minor Lab oversaw data management.

The IRRMC took shape as a public, scalable, searchable repository of data from protein diffraction experiments used to determine protein structures in the PDB in the summer of 2015, thanks in part to funding from the BD2K (Big Data to Knowledge) program of the National Institute of Health. At first, most of the contributed datasets came from large-scale projects: CSGID, SSGCID, JCSG, MCSG, and SGC. More recently, an increasing number of contributions come from individual research laboratories.

Data submitted to IRRMC are organized by projects that are composed of related data that can be processed together. A project corresponds to an individual diffraction experiment—usually, but not exclusively an experiment to determine a macromolecular structure. Projects may contain multiple datasets; e.g., multiwavelength anomalous diffraction (MAD) experiments typically have a separate dataset collected for each wavelength. Operationally, we consider as a separate dataset any series of 20 or more consecutive diffraction images (frames) occurring within the data for a project. In the data amassed by the IRRMC, the number of frames per dataset ranges from 20 to 14 400; the average is 438. The number of datasets per project ranges from 1 to 12.

**FIG. 1.**
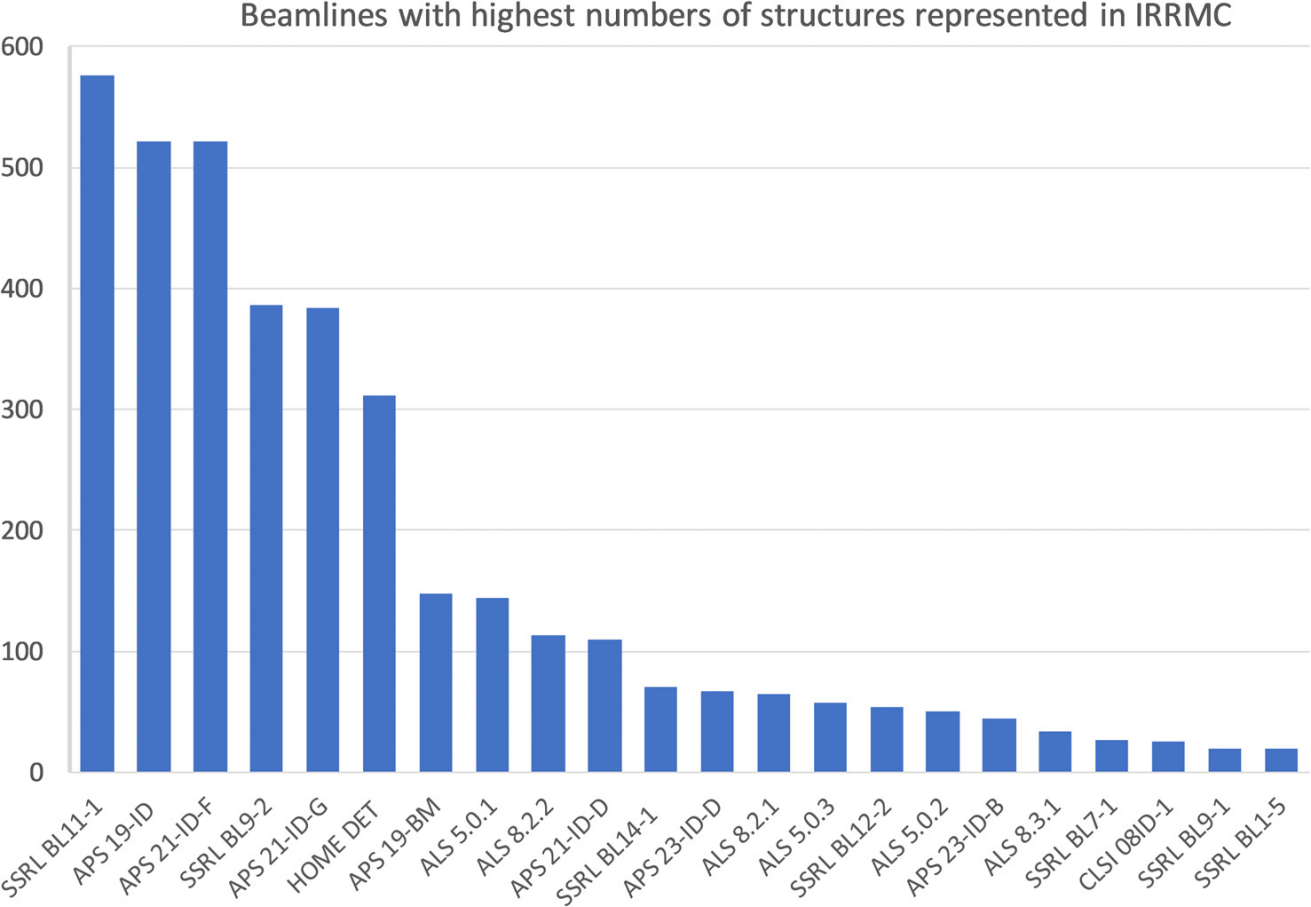
Synchrotron beamlines with the highest number of structures represented in IRRMC.

Most of the IRRMC projects are associated with X-ray structures deposited in the PDB. However, some of the projects represent beamline calibration data or “synthetic” simulated data generated to illustrate challenges of combining multiple-crystal experiments.^26^ Some projects correspond to data from multiple-crystal, multiple-ligand experiments using the Pan-Dataset Density Analysis (PanDDA) approach.^27^ As of July 2019, 55 different synchrotron stations (beamlines) from 16 synchrotron facilities were represented (see Fig. 1).

## III. IRRMC FROM THE USERS' PERSPECTIVE

Since its inception, IRRMC has strived to make the usage of the resource as simple and painless as possible. Most of the features, such as browsing, searching, and downloading, do not require registration. Data downloaded from the IRRMC may be freely used under the Creative Commons license CC0 (Public Domain Dedication Waiver).

Data submission does require using a registered account, but registration is instantaneous and requires only basic data (name, email address, and affiliation) and passing a captcha test (to prevent robots from creating accounts). An alternative method is to log in using ORCID credentials, which is facilitated via ORCID's website. Signing in through ORCID automatically creates a user account on IIRMC associated with the ORCID id. This will be distinct from an account created manually through the site.

The IRRMC interface provides several ways to access diffraction data. Recently published datasets appear at the bottom of the home page to keep frequent users up to date. At the top of the page, there is a universal search box that searches various parameters, keywords, or combinations of these. The most straightforward search is to look for the data associated with a particular PDB deposit, by simply searching for the PDB id. A variety of other text searches are available, including the project name, the PDB title, the DOI, the protein name, the structure's authors' names, structural genomics centers, beamlines, SG selection justification, synchrotron, and detector. Tags can be added to projects, and these tags can be used as search terms. For example, the tag “workshop” has been added to six datasets that are often used for demonstrations. The resolution, R, and Rfree of the projects can also be used as search terms using the comparison operators “=,” “<,” and “>.” For example, the search phrase “resolution < 1.75” currently returns 1104 results. Searches can be combined using the Boolean operators “AND,” “OR,” and “NOT” (in all caps) and grouped with parentheses. Spaces in text queries are assumed to be AND.

When a search is performed, the results are presented along with two graphs that compare the search results to all data in the IRRMC. One graph is a histogram showing the distribution of structures in resolution bins, and the other graph is a scatterplot of Rfree vs resolution. For both graphs, the data for the search results are red and the results for other data in IIRMC are in blue. The results are presented with the projects names and any tags that have been associated with the dataset contained within a light gray box. Clicking within the box will expand it to reveal additional information, including a thumbnail of the first image, a thumbnail of the structure (if one exists), as well as the structure's first author, the resolution, and the R values. A link on the right will display more detailed information about the dataset. The download link displays the size of the dataset and will open a system dialog to download the BZIP2 compressed file containing the data. Links to the deposit in the Research Collaboratory for Structural Bioinformatics (RCSB) PDB website and to the DOI information at Data Cite are also provided.

The dataset's detail page includes more information about the structure, including authors and unit cell dimensions. Projects can have more than one dataset associated with them, and the information about each dataset includes the number of frames, the crystal to detector distance, the oscillation width of each frame, X-ray wavelength, and collection locations.

Information about datasets is also accessible from PDB websites. The link on the RCSB's PDB page can be found in the “Experimental Data and Validation” section of the structure summary (the main) page of a structure (as a link to the DOI). On the PDBe site, the link is found in the “Experimental raw data” box (with a link, number of datasets, and total size).

The data submission process is straightforward. Files for submission can be dragged into the upload area on the page or selected using a system dialog box that will open if you click in the upload area. Files should be tarred and compressed using the bzip2 or gzip format. The “Submit Data” button will take you to a page that presents three categories of submission. The “Structural Genomics” and “PDB” categories are for data associated with PDB deposits and will link the IRRMC projects to the appropriate PDB entry. The title and author information for the datasets will be taken from the PDB entry. The main difference between these two categories is that structural genomics files should include the target id as a part of the file's name. The “Other” category is for files that are not directly associated with a PDB entry. These can be unsolved datasets, calibration data, or any other images that should be preserved. By uploading data to the IRRMC, the user grants the resource the right to archive, annotate, and curate the data and to make it available for download by the public. The user also acknowledges that the usage of the data will be governed by the Creative Commons license CC0.

Once data have been uploaded, they will appear in the user's profile page, along with some pertinent information such as the deposited filename, dataset size, and the assigned DOI. Collection details and other metadata will be imported from the PDB if available; thumbnail and structure image will be generated automatically. Contributors of datasets from the “Other” category need to supply some of the information that is usually extracted from the PDB, such as the project name and authorship.

Data submitted are not instantaneously available on the website because a certain amount of manual processing and validation is required. Images associated with PDB structures that have not been released will not be publicly available until the PDB deposit is released (unless the user notifies us otherwise). Accommodations can be made for users who would like to submit a large number of datasets through a protocol other than the website.

## IV. VALIDATION OF DATA AND METADATA

Validation of the data, metadata, and relations between the submitted datasets and PDB deposits is crucial to maintain the integrity of the IRRMC data. One of the validation procedures is to reprocess the diffraction data “de novo” and compare the results against the associated PDB model and metadata. This procedure ensures that the correct dataset was submitted and is linked with the proper PDB deposit. The reprocessing of diffraction images requires limited, but accurate metadata from three categories: (A) image file format, detector size and potential binning, and compression schema are necessary to read the data; (B) detector pixel size, detector orientation and the origin, and beam position are essential to properly index reflections, and (C) goniostat geometry and rotation axes relative to the detector and relative oscillation angles are necessary to meaningfully integrate and scale the data. In most cases, the metadata from category A can be automatically inferred from the image files, even if these metadata are not explicitly or accurately stored in the header (except compression schemas). The metadata from categories B and C are detector and beamline/source specific and in the case of beam position can significantly vary over time.^23,28^ This information is usually stored together with the diffraction images at the time when they are produced. In practice, the format, content, and accuracy of these metadata are subject to frequent changes and are often not documented. The correctness of this information is critical for data processing; however, we have encountered many errors, especially in older datasets.

Because of the limited number of synchrotron beamlines where macromolecular diffraction experiments are performed, it is feasible to create a database of the time-dependent basic metadata related to the diffraction experiments originating from a specific beamline: detector type, detector orientation, and goniostat configuration. For some beamlines, the beam position stored in the headers of the diffraction images is imprecise, wrong, or stored using different conventions. To account for such cases, we have created a database of the historical beam positions collected from information published by beamlines, recovered from the previous experiments in our laboratory or based on reference datasets from these beamlines that were processed manually. Moreover, we have observed the detector serial number present in the diffraction image header is a more reliable way to identify the beamline used for a dataset than the information contained in a PDB deposit. This has the added benefit that the datasets can be processed even without relying on the associated PDB deposition. Even at this point, we have discovered several discrepancies indicating that the deposited data were not the original data used for structure determination of the particular deposit. The basic metadata validation is prerequisite for automated processing of the datasets.

We have created a pipeline for automatic reprocessing of diffraction data using the automatic mode of the HKL-3000 structure determination suite.^29^ For testing purposes, the pipeline was run on 3584 IRRMC projects with structures that were released by the PDB (see Table I). Of these, 1072 were originally solved by MAD, 737 by single-wavelength anomalous diffraction (SAD), 1694 by molecular replacement (MR), and 81 by a combination of these methods (e.g., MR/SAD) or another method, e.g., multiple isomorphous replacement (MIR).

**TABLE I.** Summary of automatic reprocessing and structure redetermination.

	MAD	SAD	MR	Other	Total
Attempted reprocessing	1072	737	1694	83	3584
Successfully reprocessed	1037	701	1592	74	3404
Same space group and cell as in PDB	942	596	1291	57	2886
Same space group and cell, same or better resolution as in PDB	503	282	637	25	1447
Successful automatic phasing by SAD	730	505	…	…	1235

**FIG. 2.**
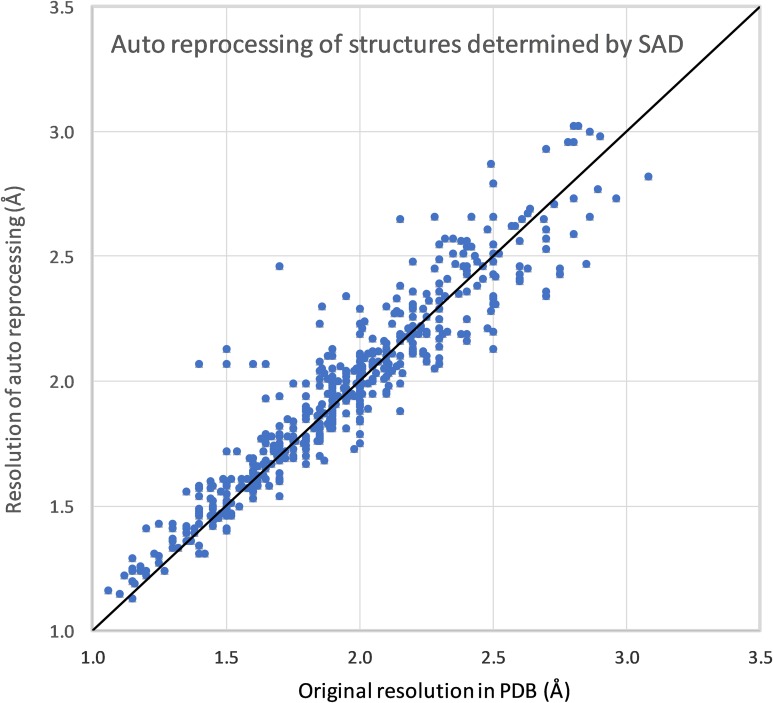
Resolution achieved by automated reprocessing of data vs the resolution in the original PDB deposit for structures determined by SAD.

**FIG. 3.**
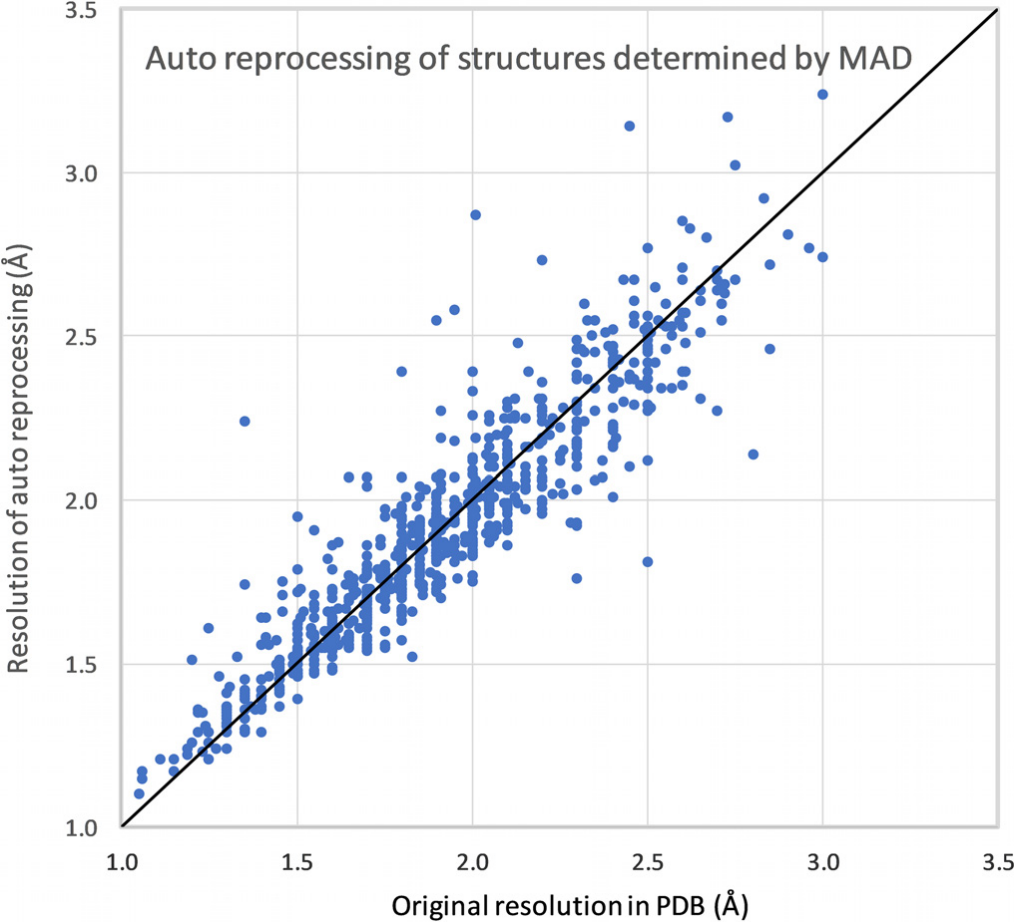
Resolution achieved by automated reprocessing of data vs the resolution in the original PDB deposit for structures determined by MAD.

The pipeline was run iteratively—projects that failed during one iteration were grouped by beamline and time of the experiment and analyzed. If the processing failure resulted from missing or incorrect metadata, then the metadata were corrected. In about 5% of cases, automatic processing could not proceed due to various problems with data and/or metadata that we were not able to remediate—e.g., some part of the data was missing, missing calibration files for some old detectors, etc. For the other 95% (3404 projects), automatic processing was successful, i.e., proceeded through indexing, integration, and scaling without abnormalities such as unreasonably small unit cell dimensions, unrealistic mosaicity, or abnormally high R-merge/R-meas/R-pim. For 81% of the cases (2886 projects), automatic processing resulted in the same space group and cell dimensions as in the original model in PDB. The resolution of the reprocessed data (as determined by I/sigma = 2.0 in the highest resolution shell) was generally close to the resolution reported in the original model in the PDB (see Figs. 2 and 3). For about half of the automatically reprocessed projects (1447 cases), the processing yielded the same or better resolution as the resolution in the original PDB deposit, with an average improvement of 0.1 Å.

For projects originally solved by MAD or SAD, we used HKL-3000 in auto mode to perform automatic structure redetermination (without rerefinement). For the projects originally solved by SAD, this approach succeeded in 505 out of 701 cases (72.0%); it failed in a number of cases with low completeness or where data were twinned. For projects originally determined by MAD, we conducted a computational experiment to examine the feasibility of solving the structure by SAD using the data collected at the wavelength with the largest anomalous signal. Out of 995 MAD projects, this approach succeeded in solving 730 (73.4%); most of the failures could be attributed to insufficient completeness of the selected dataset. SAD structure solution for structures originally determined by MAD also failed for some projects with a high number of molecules when the number of copies in the asymmetric unit was off, resulting in an incorrect number of peaks for peak search.

## V. USAGE OF DIFFRACTION DATA FROM PUBLIC REPOSITORIES IN IMPROVING PREVIOUSLY DETERMINED PROTEIN STRUCTURES: CASE STUDIES

In our work on various projects within the last several years, we have encountered multiple PDB entries with significant problems in them. Together with collaborators from other labs, we have rerefined some of these structures following recently published state-of-the art guidelines.^30^ We have always tried contacting the original authors to discuss introduced changes and, if an agreement was reached, deposited the corrected structures in the PDB to replace the original entries as joint deposits with the original authors. In multiple cases, these contacts resulted in further improvement of the structures via very productive collaboration.^11,31,32^ When working on these structures, we have always requested the original diffraction images, which, if available, often provided significant advantages in structure analysis and reinterpretation. These improvements were due to improvement of data quality and resolution by processing with updated software,^29^ the exclusion of bad images (e.g., due to severe radiation damage), or the extraction of the anomalous signal, which can be extremely useful for the accurate identification and modeling of transition metals, halogens and halogen-containing ligands, and even potassium and calcium ions and sulfur- and phosphorous-containing ligands.^33,34^ Unfortunately, more often than not it was impossible to obtain the images as described in the examples below. In addition, we have participated in or heard about several anecdotal stories in which a collaborator or an acquaintance could not locate the original images that were needed, for example, for an update to a PDB deposit requested by a reviewer of a journal to which the respective structural paper was submitted. Reasons for inability to recover images included a hard drive failure, the responsible person leaving the lab, and even the images not being timely retrieved from the synchrotron beamline where they were collected.

In our recently published paper,^11^ we rerefined 10 structures of metallo-β-lactamases deposited to the PDB previously by other groups. Rerefinement of these structures following our published guidelines^30^ resulted in various improvements of maps and models. Unfortunately, the diffraction images were obtained only for two of the 10 structures. One of them, 4hky, was solved as a part of structural genomics effort, and the images were accordingly stored at the IRRMC. The reprocessing of these images and subsequent rerefinement resulted in an extension of resolution limit and a drop of Rfree by 4.3%. The improved maps did not show support for the originally modeled ligand, which was substituted with an unknown ligand (UNL). In the second case, 3m8t, the diffraction images were not publicly available, but upon our request, the original depositor uploaded the images for this entry to the IRRMC. The images were reprocessed, and the resolution limit was extended from 1.33 Å to 1.20 Å by including the areas in the corners of the detector. As a result, the total number of unique reflections has increased by 21%. Importantly, the dataset was reprocessed in the anomalous mode, and the resulting anomalous maps were used to locate the zinc ions in the model, resulting in the addition of three noncatalytic zinc ions. Overall, the model was significantly improved.

For the other 8 of 10 rerefined structures, recovering the datasets proved to be impossible. Reprocessing original images might have resulted in significantly improved quality of the maps, as I/sigma in the last shell is as high as 19 for some of these entries. For example, a PDB entry 4nq7, which was deposited in the PDB in 2013, did not have sufficient evidence for placing a bound inhibitor in a proposed location, even though its presence at a low occupancy could not have been excluded after the consideration of POLDER maps.^35^ Data reprocessing could have improved the maps, potentially resulting in a more confident interpretation in this or that way. In addition, the entry lacked crucial data processing statistics with multiple NULL entries. Unfortunately, neither the original diffraction images nor the data processing log files could be located. Therefore, the ligand was replaced with an unknown atom (UNX), and the structure was redeposited with the same limited scaling statistics as the original one. These examples show how even recently deposited structures may lack crucial experimental data and metadata.

Two other examples from the same paper,^11^ PDB entries 1jt1 and 1k07, were deposited much earlier—in 2001. These structures were dramatically improved by the rerefinement (e.g., a drop in Rfree by 3.5 and 4.0%), which can be mostly attributed to the dramatic improvement of the refinement, model-correction, and structure-validation software since the original structures were deposited almost two decades ago.^30^ For one of the structures (1jt1), the rerefinement clearly showed that there is no evidence from the experimental data for the placement of the ligand near the active site as in the original deposit. Despite these improvements, the electron densities in the active sites of both structures were not conclusively interpretable, and it was concluded that a set of unknown atoms is the safest interpretation for both structures. Data reprocessing might have resulted in better maps, e.g., due to potential extension of the resolution as I/sigma in the last resolution shells for these two structures was equal to 8.4 (1jt1) and 19 (1k07) and/or due to better processing with the improved software. The authors of the original deposits did preserve the magnetic cassettes with diffraction images. Unfortunately, no feasible way of restoring images from these cassettes was found. This story illustrates that even if a lab makes the best effort to preserve the original data, a public repository seems to be a much more reliable and useful resource.

When conducting an overview of GHRA and GHRB crystal structures in the closed conformation with a ligand bound in the active site,^36^ we have encountered a GHRB protein structure with an incorrectly identified ligand in the active site (5aow). The rerefinement with the deposited structure factors clearly revealed the identity of the ligand, which happened to be malonate that was used at 1.7 M concentration in the crystallization solution. However, data reprocessing could have further improved the structure. Unfortunately, the authors of the structure did not locate the original images, even though the original structure was deposited recently (2015).

Similarly, a software tool for automatic recognition of ligands in electron density by machine learning^32^ has revealed multiple structures with incorrectly modeled ligands. We have rerefined four sample structures, discussed the changes with the original authors, and deposited all four corrected structures in the PDB to replace the original entries. Data processing statistics indicate (e.g., I/sigma equal to 2.9 in the last shell) that some of these structures could have been further improved if the diffraction images were available.

## VI. CONCLUDING REMARKS

The results presented here demonstrate that a great deal of the information necessary for the determination of a protein structure is preserved in the archived X-ray diffraction data. In most cases we encountered, the metadata missing from diffraction images can accurately be reconstructed, even for old depositions, by the manual annotation and comparison to diffraction experiments from the same beamline and similar time period. We are developing a mechanism to share the metadata essential for processing the images.

We are continuing to develop the pipeline for validation of the data and metadata by the automated processing of the submitted datasets. We plan to add full structure solution using Fourier synthesis and MR to check the agreement of the original PDB deposit with the structure factors generated after automated processing of raw diffraction data. We also plan to check correlation of the structure factors resulting from the reprocessing with the structure factors deposited to the PDB. However, full analysis and interpretation of the discrepancies between the reprocessed data and the original data deposited to the PDB would require additional information beyond diffraction data—namely, what computational procedures have been applied by the authors of the original PDB deposit. We suggest that such detailed information on computational protocols used for structure determination and refinement in the course X-ray crystallography workflows should be preserved and shared via public repositories. Without this detailed computational protocol, it is not always possible to reproduce the exact results, as demonstrated by the differences between the resolutions presented here that can stem from a variety of reasons, such as different resolution-cutoff criteria, differences in software and parameters, or unlikely mismatch between deposited raw diffraction data and the deposit.

Even though the recent spectacular successes of cryogenic electron microscopy (cryo-EM) have led some scientists to question the future of X-ray crystallography as a widely used method of protein structure determination, in our opinion, X-ray crystallography will continue to play an important role in structural biology in the foreseeable future. In fact, we expect that the volume and complexity of X-ray diffraction data produced on protein crystallography beamlines and home sources throughout the world will continue to increase, requiring a corresponding scaling up in diffraction data repositories including the IRRMC.

All of the current diffraction data repositories, including the IRRMC, will need to be extended to efficiently handle data produced by X-ray crystallography techniques applicable in structural dynamics studies, such as X-ray free electron laser (XFEL) and serial crystallography. No repository currently accommodates datasets corresponding to “structural movies” in addition to sets of diffraction images. Datasets produced by experiments using time-resolved protein crystallography may put a significant strain on the resources required of a data archive. The resources required are related more toward the efforts necessary to expand the functionality rather than the purchase of new equipment, although the expansion of storage capacities may also be necessary.

The widespread use of data archives that support open access and efficient searches of data generated by various biomedical imaging technologies is envisioned as a potential driver of the “next revolution” in biomedicine.^37,38^ However, to be truly useful to scientists, data resources gathering images, such as the IRRMC, cannot operate in isolation, but rather form part of a larger “data ecosystem.” Development of tools that allow data discovery across multiple resources, such as the Datamed^39^and the Google Dataset Search,^40,41^ will be essential for this ecosystem of open data resources to function properly.
